# Skeletal growth and development dictate the processes of vertebral fracture in the pediatric spine; a review emphasizing fracture biomechanics of the vertebral body during the period of skeletal immaturity

**DOI:** 10.3389/fped.2025.1468926

**Published:** 2025-05-08

**Authors:** John G. McMorran, Diane E. Gregory

**Affiliations:** ^1^ Department of Kinesiology and Physical Education, Wilfrid Laurier University, Waterloo, ON, Canada; ^2^ Department of Health Sciences, Wilfrid Laurier University, Waterloo, ON, Canada

**Keywords:** vertebral fracture, endplate fracture, ring apophysis fracture, pediatric spine fracture, pediatric spine biomechanics

## Abstract

Infancy, childhood, and adolescence involve changing body proportions, muscular strength, and the complex processes of skeletal growth, contributing to a unique subset of biomechanical considerations when vertebral fractures result from falls from height, motor vehicle accidents, nonaccidental injuries, and sport and manual labour. In this review, the biomechanics of compression fractures, burst fractures, seatbelt syndrome, nonaccidental trauma, defects of the vertebral endplate, and ring apophysis fractures are all detailed regarding their manifestation in the pediatric spine. Interactions between pediatric diseases, the intervertebral disc, and the spine's facet joints are also briefly discussed, lending additional context toward the unique etiologies of pediatric vertebral fracture. The present narrative review seeks to provide a detailed overview of the key relationships responsible for the unique biomechanical considerations governing vertebral and endplate fracture, in the pediatric population.

## 1 Introduction

In the human lifespan, significant growth of the spine occurs throughout the periods of infancy and adolescence, where both the anatomy and the material properties of the spine undergo considerable changes. Vertebral fracture represents a significant orthopedic injury at any age, but the distinct characteristics of the growing spine result in several important and unique biomechanical considerations with regard to the etiology of pediatric vertebral fracture injuries.

The purpose of the present paper was to perform a narrative literature review of relevant biomechanical research pertaining to how development of the spine during infancy/early childhood, and growth of the spine during adolescence, contribute to the heterogeneity of the vertebral fracture process across these age ranges.

## 2 Methods

Narrative literature review methods identified relevant peer-reviewed research papers through extensive keyword searches on Google Scholar, Pubmed (Medline), and Wilfrid Laurier University's library search engine. The abstracts for articles possessing key words in their titles relevant to the present topic were reviewed. Abstracts discussing pediatric vertebral body (including vertebral endplate) fracture biomechanics, or pediatric vertebral body (including vertebral endplate) fracture patterns, were selected for incorporation in the present review. Notably, several other topics (contributions of pediatric disease states, the biomechanical contributions of the intervertebral disc and facet joints) are also included. This review does not provide a comprehensive overview of the former topics listed, but instead uses them to contextualize discussion of the biomechanics of vertebral body fractures. For this review, “pediatric” refers to all life stages before skeletal maturity.

### 2.1 Keywords

The following search terms were used to define bony fractures of the spine:

Vertebra(e,l) fracture; Spine fracture; Endplate fracture; Ring apophysis fracture/avulsion/growth plate/separation/limbus fracture/slipped vertebral epiphysis; Biomechanics; Fracture Mechanics.

Articles with a focus on biomechanics in the infant and/or child and/or youth and/or adolescent and/or juvenile spine were included in the review.

## 3 Vertebral fractures in the infant/child spine

### 3.1 Morphological factors that contribute to vertebral fracture

The infant spine possesses upwards of 130 active growth plates (1), and is only approximately 30% ossified during infancy (2). During infancy, childhood, and adolescence, the vertebral body grows axially through endochondral ossification, similar to long bones (3, 4). Axial growth occurs via unidirectional expansion of the superior and inferior growth plates outward from the vertebral centrum, while radial expansion results from the apophyseal growth plates encircling the vertebral body (3, 4). Two primary growth centers, the neurocentral synchondroses, contribute to the development of the vertebral arch, from which the facet joints also arise (i.e., from the vertebral pedicles) (3, 4). The endochondral growth plates lie beneath the cartilaginous vertebral endplates (EPs), which vary in thickness across the vertebral body (5, 6). The EP is thinnest at the center, facilitating nutrient exchange with the nucleus pulposus (NP), while the annulus fibrosus (AF) integrates with the EP toward its mid-point and periphery (5–10). Additionally, the articular cartilage of the facet joints minimizes friction and protects the underlying bone (3, 4). Last, vertebrae possess a cranial-caudal heterogeneity regarding cortical bone density, and the trabeculae underlying the vertebral EP. Multiple investigations have demonstrated a greater porosity/reduced density in the cortical bone of the cranial vertebral EP vs. the cortical bone of the caudal vertebral EP in the same vertebral body, and also reduced trabecular density and thickness in the cranial vs. caudal regions underlying the vertebral EPs (11–13).

During growth, the vertebral body undergoes significant morphological changes. In newborns, vertebral bodies appear convex (14), but by the second year, the endplates (EPs) are typically described as square or flat in the sagittal plane (14). In adults, both cranial and caudal EPs become concave, curving inward toward the centrum of the vertebra (14–18). These age-related changes in EP geometry reflect the substantial structural adaptations occurring from infancy through young adulthood, with increasing concavity persisting into adulthood (14–17, 19). The transition from flat EPs in pre-ambulatory infants to concave but unfused EPs in ambulating adolescents and young adults may indicate a period of EP-mediated vertebral body weakness.

An experiment by Amonoo-Kuofi et al. (15) analyzed lateral radiographs of the lumbar spine in a cohort of 615 patients (310 females, 305 males) ranging from 10 to 64 years in age and found a positive relationship between age and increased vertebral body concavity described as a greater convexity (index of wedging) of the IVD. However, the greater EP concavity that accompanies aging does not occur linearly, and Amonoo-Kuofi et al. (15) describe the process as alternating between periods of “overgrowth” and “thinning” throughout the lifespan. A finite element model constructed by Meijer et al. (20) modelled the changes in vertebral dimensions that accompany the period of adolescent growth in the human spine. Their finite element model demonstrated that biomechanically, deeper EP concavities resulted in increased mechanical stiffness in a spinal segment.

Similarly, during their *in vitro* experiment, Dudli et al. (21) reported that under high-rate impact loading, specimens with deeper vertebral EP concavities required more mechanical work and achieved a higher overall load before failure. The experiment conducted by Dudli et al. (21) employed *in vitro* rabbit motion segments as their vertebral fracture model, so further work must be conducted to confirm such a relationship in the skeletally immature human spine. However, due to the significant changes in vertebral EP concavity that occur with growth, the specific mechanical considerations leading to vertebral and EP fracture seem to be significantly impacted by these alterations in vertebral body and EP geometry (20).

Compression fractures are the most commonly observed vertebral fracture pattern in pediatric medicine (22–26), occurring in both healthy and pathological conditions (27). In their cohort of 165 patients (ages ranging from 10 months to 17 years), 78% of fractures were diagnosed as “compaction” (i.e., compression) fractures by Compagnon et al. (23), with only 8% of fractures diagnosed as burst fractures, 2% as Chance fractures, 1.21% as odontoid fractures, and the remaining 20% categorized as “other” lesions (23). The affected spinal level varies with skeletal maturity. Cervical spine fractures are more common in younger children, while lumbar spine fractures occur more frequently in older children and adolescents (28–32). This pattern may be influenced by injury mechanisms, changing body proportions, musculoskeletal development, and increased athletic involvement in older age groups.

### 3.2 Impact of disease on vertebral fracture

Various diseases also influence vertebral fractures in pediatric population. Taylor et al. (27) conducted a retrospective review of 181 patients diagnosed with vertebral fractures before age 18, excluding fractures caused by trauma. Among these atraumatic cases, 15% had primary osteoporosis (7% idiopathic, 7% osteogenesis imperfecta, 2% other connective tissue disorders), while 65% had secondary osteoporosis, primarily due to immunosuppressant treatment (46%), acute lymphoblastic leukemia (10%), malabsorption (7%), or immobility (2%).

In a cross-sectional study conducted by Halton et al. (30), 186 pediatric patients (age range 1–17 years) were examined at approximately the time of their initiation of chemotherapy for acute lymphoblastic leukemia, for bone densitometric and morphometric variables; these variables were then compared against normative age and sex-matched data, originating from the CDC's National Center for Health Statistics database. Halton et al. (30) compared *Z* scores for chronological age as well as “bone age” (this variable served as an approximation of skeletal maturity) between their patient cohort and normative data from the database. Interestingly, Halton et al. (30) reported that their patient cohort had a greater mean height compared to the control data. But, despite leukemia patients having a greater average height, Halton et al. (30) reported significantly reduced bone mineral density *Z* scores for this population. Among this population, Halton et al. (30) reported fractures affecting 16% of patients. Even more interestingly, when comparing within the cohort of pediatric leukemia patients, Halton et al. (30) reported that the pediatric patients diagnosed with a vertebral fracture also had lumbar spine bone mineral density *Z* scores 1.0 standard deviations below the *Z* scores of pediatric leukemia patients unaffected by vertebral fracture. Unsurprisingly, 55% of vertebral fracture patients suffered from back pain, while only 20% of the pediatric leukemia patients without vertebral fracture suffered from back pain. Halton et al. (30) reported that, for every standard deviation reduction in lumbar bone mineral density, vertebral fracture risk increased by 80%, and furthermore, that lower *Z* scores for lumbar bone mineral density were associated with more severe (i.e., greater reductions in vertebral body height) vertebral body fractures overall.

As a solitary variable, bone mineral density is inadequate to fully predict the mechanical competency of the human skeleton, and this remains true for pediatric patients at-risk of vertebral fragility fractures resulting from either primary or secondary osteoporosis (33–35). For this reason, the presence of an atraumatic vertebral body compression fracture in a pediatric spine—or a history of atraumatic/low energy fractures at other skeletal sites—regardless of the patient's quantified bone mineral density *Z* score, should serve as a positive diagnostic sign of the presence of pediatric osteoporosis (35), where diagnosis of a vertebral body fracture should prompt further investigation into the underlying cause of the atraumatic vertebral fracture observed.

Radiographically, distinguishing normal pediatric vertebral growth from injuries, pathological conditions, or abuse-related trauma can be challenging, especially when the injury mechanism is unclear. Jaremko et al. (36) provide an excellent review distinguishing between growth-related radiographic abnormalities, and fracture patterns associated with trauma and disease in the pediatric spine. Given the complexities of a growing spine and the potential for underlying conditions to contribute to the pathomechanics of injury, clinicians must be familiar with common normal radiographic variants of the pediatric spine, to accurately differentiate these from true injuries.

In addition to vertebral and EP morphological changes, in the infant spine, the IVDs are also uniquely morphologic and at their most hydrated due to a high concentration of hydrophilic proteoglycans in the NP (37–40). This high degree of water retention results in a more viscous NP response under axial loading, influencing both EP and vertebral fracture mechanics (41–43).

### 3.3 Biomechanics of vertebral fracture in the infant spine

#### 3.3.1 Etiology of burst fractures

Viscoelasticity refers to the phenomenon where increasing velocities of loading increase the mechanical stiffness of a material; bone itself exhibits this mechanical property (44). In infancy, the spine exhibits its most viscous response due to high levels of tissue hydration. This viscoelastic interaction between highly hydrated tissues and the rapid application of mechanical forces drives the burst fracture etiology.

**Figure 1:**
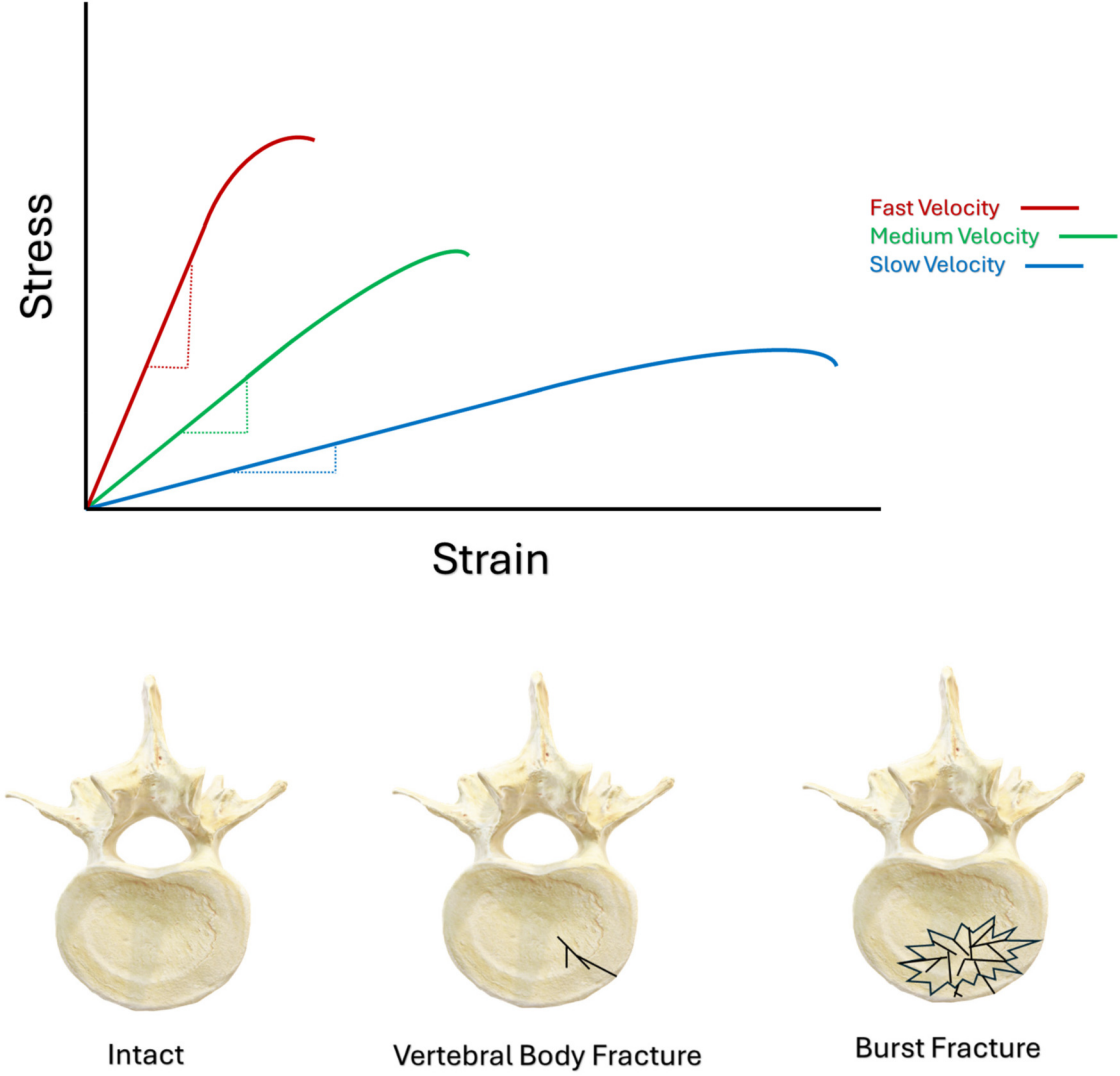
Schematic illustrating the leftward shift of bone's stress-strain curve, stemming from increased velocities of loading. Schematic illustrating axial view of the different morphology between an intact vertebral body, a simple vertebral body fracture, and a burst fracture.

Etiologically, burst fractures result when the rapid application of axial compression to a vertebra generates a shift in the stress-strain curve (Figure 1, Image 1) resulting in the development of supraphysiological forces beyond the mechanical competency of the vertebral body. Under circumstances of rapid loading, the vertebra has very little time to disperse the applied energy. This results in a rapid accumulation of mechanical energy far beyond the mechanical competency of the vertebral body, such that catastrophic fracture ensues. Furthermore, the mechanical toughening responses of bone itself have been shown to significantly diminish when bone is loaded at greater velocities (45–47). The toughening mechanisms of bone are a product of the geometry of bone tissue, and its material properties (45–48), both of which are subject to age-related variations over the course of the human lifespan (49–51). Velocity, geometry, material properties, and fracture orientation (Mode I, II, III, or mixed-modes) of loaded bone tissue will all interact with the process of fracture (45–53), altering the predominant processes of failure, depending upon the balance of micromechanical factors involved in the loading event (52, 53).

IVD hydration also significantly contributes to the burst fracture etiology. The deformation characteristics of the NP are significantly influenced by the rate at which it is loaded (54), and this in-turn will interact with the forces exerted by the NP onto the vertebral EP. Specifically, compression of an IVD's NP between adjacent vertebrae increases the hydrostatic pressure in the NP, and the more rapidly this occurs, the stiffer the NP's response will be (54, 55). As such, this massive amount of accumulated energy will be released by the vertebral body in a very short period of time, by the creation and propagation of multiple fracture planes. The reduced ability for cortical and cancellous bone to arrest propagating fractures at high velocity results in a significantly greater potential for fracture propagation than when velocities are slow (45–47). The result is a comminuted or “burst” fracture pattern, capable of projecting shards of bone into the spinal canal, (Figure 1, Image 2). *In vitro* biomechanical investigations using human cadaveric tissue (56–63), *in vitro* animal models (64–68), and finite element investigations of the burst fracture process (64, 69), consistently report greater vertebral body comminution, and greater degrees of canal encroachment by bone fragments during higher velocities of loading. Experiments investigating the burst fracture process tended to apply the greatest magnitudes of kinetic energy to spinal segments under the fastest conditions of loading (56–69). Therefore, the combination of both the overall magnitude of kinetic energy applied to the spine, along with the velocity of its application, appear to drive the burst-fracture etiology towards more severe states of vertebral body comminution.

Another contributing mechanism to burst fracture etiology appears to be posture. *In vitro* research has noted that burst fractures tend to manifest at the apex of a spinal curve loaded in rapid axial compression. Specifically, three and five segment motion segments, (both human cadaveric and animal models) are consistently reported to be placed into some degree of anterior flexion, resulting in consistent reports of the central, apical vertebra being the most commonly injured (56, 57, 60, 61, 63, 66).

Translating this towards the etiology of human injury, it would be anticipated that the segment located at the apex of the spinal curve during the rapid loading resulting from a head-first or sacrum-first fall, would be the site of greatest vulnerability to failure via the burst fracture mechanism. The spine possesses natural kyphotic and lordotic curves and can also achieve significant curvature through changes in sagittal posture. As such, the apical vertebrae in a spine will depend on the posture of the spine at the time it is loaded, and can change depending upon the state of extension, flexion, or combination of these curves in the rapidly loaded spine.

Conversely, in a cross-sectional study examining 152 patients and a total of 169 burst fractures, Bensch et al. (70) reported that burst fractures most commonly occurred at the thoracolumbar junction and between T5 and T8 in both males and females. These authors proposed that the high incidence of thoracolumbar and cervicothoracic burst fractures coincided with the most mobile spinal region, while the common finding of burst fractures in the T5–T8 spine region, coincided with fractures in the most rigid spinal region (70); these findings illustrate the disparity in mechanical circumstances throughout different regions of the spine, all of which can result in a burst fracture manifesting in the vertebral body.

Another significant biomechanical consideration is the thoracolumbar junction. The ribcage's articulation with the spine ceases at the T12 level (71), and the transition from the rigid thoracic curve to the flexible lumbar curve may predispose the thoracolumbar vertebra to a unique combination of mechanical forces. The anterior concavity of the thoracic kyphosis typically transitions to the posterior concavity of the lumbar lordosis proximal to the thoracolumbar junction. The meeting of these two oppositely orientated curves subjects the transition vertebra(e) to significant bending forces in conjunction with longitudinal compression resulting in mechanical vulnerability to vertebral fracture in this region. This observation is consistent with the propensity for burst fractures to occur in the thoracolumbar junction *in vivo* in equal distribution in both sexes, by a variety of mechanisms of injury including motor vehicle collisions (MVCs; including automobiles and motor bikes), falls from height, and sporting incidents (70).

Ultimately, the micro-mechanics of the burst fracture entity entail the rapid accumulation of mechanical energy, far beyond the mechanical strength of the vertebral body. When the spine is loaded in axial compression, the trabecular network of the vertebral body is predominantly loaded along the longitudinal axis of the spine. As such, the longitudinally oriented pillars of the trabecular system will be most in-line with the primary direction of loading while the perpendicular transverse pillars experience flexural and shear forces. Not surprisingly, transversely oriented trabeculae are prone to failure by mechanisms of shear and flexure (72), while longitudinal pillars are more prone to failure via buckling (72). The vertebral trabecular architecture is a system of interdependent supports, so compromise of any individual trabecula can alter the mechanical dispersal of load in neighbouring trabeculae, potentially further compounding the mechanics of injury.

During burst fracture, the rapid loading results in a nearly incompressible NP (54). As a result of this incompressibility, further axial loading causes the concaved cartilaginous EP to bend around the incompressible NP, such that the accumulated flexural forces in the bending EP result in failure (73, 74). Burst fracture is not a mutually exclusive process between trabecular collapse and EP failure, but rather a combination of these two phenomena; Jackman et al. (73) illustrated the interconnectivity between flexure of the EP, and the mechanical contribution of the trabecular bone beneath the vertebral EP, towards resisting the intrusion of the bending EP into the vertebral body (73).

Following burst fracture, intrusion of one or more bony pieces into the spinal canal can generate neuropathic signs and symptoms. The neuropathic signs and symptoms generated by burst fracture will correlate to the level of injury in the spine, and the severity of spinal cord injury. Importantly, Wilcox et al. (64) demonstrated that the site of vertebral fragments during radiography does not adequately represent the maximum intrusion achieved by vertebral fragments during the burst fracture event. Therefore, burst fracture generated neuropathies cannot be fully represented by the degree of canal encroachment visualized upon admission at the hospital, recommending that a more comprehensive neurological investigation should be undertaken, if burst fracture is visualized in an admitted patient.

#### 3.3.2 Rapid flexion and distraction injuries (lap belt injuries, “chance fractures”, endplate avulsions, and “shaken baby” syndrome)

Due to the high magnitude of forces involved in MVCs, and the restraint of motor vehicle occupants via seatbelts, MVCs create a specific set of conditions where lab belt injuries, and Chance fractures can manifest in the spine.

During a head-on collision, a vehicle decelerates at an exceptionally rapid rate, and seatbelts will lock to secure passengers in their seats. If a vehicle is stationary and is struck from behind by a rapidly moving vehicle, the resulting acceleration of passengers can be arrested by seatbelts as well. By both mechanisms, the momentum of passengers' bodies will interact with the restraint of the seat belt across their lap and obliquely across their torso. Issues arise when seat belts are improperly worn by passengers, or when children of inadequate height sit in adult passenger seats. There is also a high correlation between abdominal hematoma and/or lesions of internal organs, accompanying severe flexion and distraction injuries in the spine (75–77). As such, abdominal wall or intraabdominal organ hematoma may be useful clinical indications that the spine should also be investigated for trauma.

**Figure 2:**
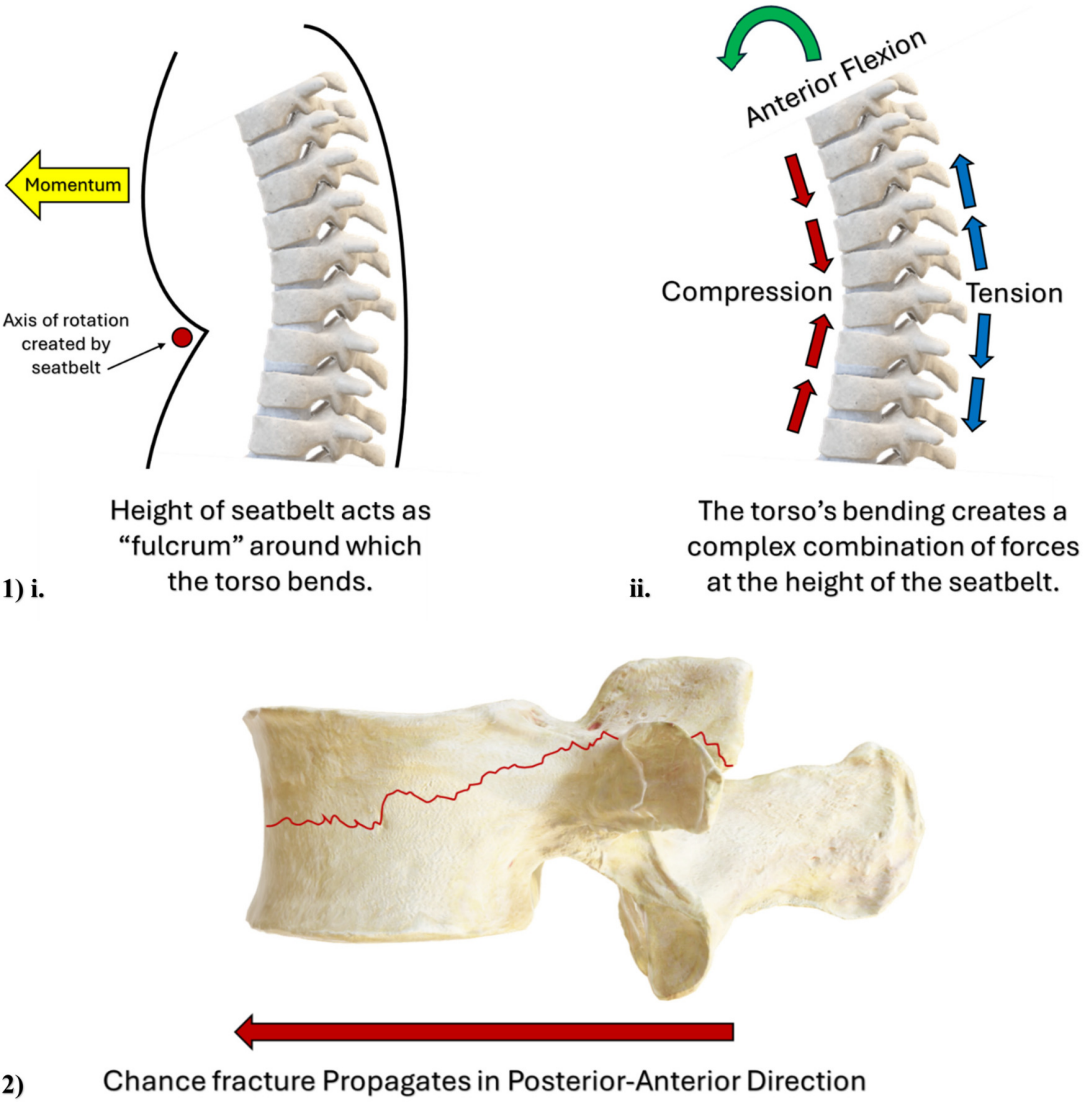
(Image 1) schematic illustrating the “fulcrum” effect of the seatbelt on a motor vehicle passenger's torso, and the complex combination of biomechanical forces acting on a motor vehicle passenger's spine. (Image 2) Schematic illustrating the direction of Chance fracture propagation from the posterior-to-anterior direction.

Lap belt injuries occur when a single point of anchorage is applied to stop the occupant's ejection from their seat, namely the lap belt (75–78). The sudden locking of the lap belt will halt the body's anterior translation from the seat at this single point of anchorage, and without the assistance of the torso-restraint, the upper body will translate anteriorly away from the back of the seat. As such, the anteriorly directed momentum of the upper body against a locked lap belt will act like a fulcrum around which the torso will rotate (75–78); (schematic in Figure 2, Image 1, i). The location of this fulcrum can vary, as a seatbelt is situated lower down on a passenger of adult stature than if a child of inadequate height sits in the same seat. Anterior translation and flexion of the passengers' torso can result in significant, and complex combinations of flexion, compression, and distraction forces acting on the vertebral body, (Figure 2, Image 1, ii) (75–79). Lap belt injuries carry no specific morphological appearance, named instead for the mechanism of injury by which they occur.

An improperly secured child using only the lap-belt can result in lap-belt injury, due to flexion and translation of the torso. If the torso is secured with both lap and torso belts, forward flexion and translation can only occur at the level of the head and neck, such that pediatric cervical seat belt syndrome can ensue (78). Trauma to the upper cervical levels in the form of fracture and/or fracture/subluxations is more common in younger children/infants, with cervical seatbelt syndrome more often manifesting as injury to the lower cervical levels, in older children/adolescents (80).

When significant acceleration-related forces occur rapidly about the cervical region of the spine, these injuries are sometimes colloquially known as “whiplash” injuries, except the term whiplash may be an oversimplification of two different etiologies of injury. Specifically, whiplash is best defined as predominantly a soft-tissue injury which is largely undiagnosable through imaging, especially in its milder to moderate manifestations (81–86). In addition, the predominant mechanism resulting in whiplash injury stems from the sudden and unexpected acceleration of a parked vehicle, due to a rear-impact collision (81–86). In their simulated whiplash experiment, Panjabi et al. (86) demonstrated the complex spinal kinematics of the whiplash etiology, and how it varied temporally over the course of the injury. Specifically, initial extension of the lower cervical spine gave way to a complex “S” shaped pattern during the second stage of the injury (at approximately the 50–75 ms stage), which Panjabi et al. (86) posited to be responsible for many of the symptoms generated through this mechanism. Under its current definition, whiplash injuries are most appropriately described as a sudden forward acceleration, causing soft-tissue injury (muscles, ligaments, and/or facet capsule strains or impingement), but without the magnitude of energy required to produce vertebral fracture (81–86). It seems likely that flexion/distraction fractures can arise in the cervical spine through similar kinematic patterns to the flexion, distraction, and compression that occur during the mid-to-end stages of whiplash injuries. However, in order for vertebral fracture to arise, considerably greater magnitudes of mechanical energy must be involved in the injury event, than those that tend to generate soft tissue injury alone.

Ultimately, rapid flexion disproportionately loads the anterior vertebral body in compression, unloading the facet joints and posterior vertebral body, as the body's centre of mass rotates and translates anteriorly. Akin to burst fractures, rapid flexion-mediated overload can cause vertebral trabeculae to collapse and/or vertebral EP failure, while considerable distraction forces can accrue in the facet joint capsules, posterior longitudinal ligament, and posterior IVD.

While lap-belt and whiplash mechanisms can generate a variety of injuries in the spinal column, Chance fractures (Figure 2, Image 2) and EP avulsions represent two specific morphological entities, both of which appear possible via the flexion-distraction mechanism. Chance fractures occur when flexion and distraction forces exceed the strength of the pedicles and vertebral body (24, 87–90). Flexion will elongate the posterior ligamentous structures of the spine, including the facet joint capsules, which imposes considerable tensile strain upon these structures (87). When the combination of flexion plus distraction forces intersects at the mid-height of the vertebral body, these oppositely orientated vectors of force impose an exceptionally high degree of mechanical demand on these regions. Forces in excess of the facet joint and vertebral body strength will manifest as a Chance fracture; a fracture that splits the pars interarticularis and propagates in a transverse direction, horizontally bisecting the vertebral body in the posterior-to-anterior direction (87); (schematic in Figure 2, Image 2). Mechanistically, a multicentre retrospective review of pediatric Chance fractures conducted by Arkader et al. (91) noted that 34/35 fractures in their pediatric cohort occurred due to MVC, and 1/35 originated from a “sledding” accident. This retrospective report clearly illustrates the close association between the evolution of Chance fracture in the pediatric spine, originating from a patient's involvement in an MVC, and this relationship is seemingly exacerbated by improper use of seatbelts in pediatric passengers (90).

In addition to Chance fractures, the tensile forces achieved during rapid flexion and distraction of the spine can also manifest as an avulsion of the cartilaginous EP from the cortical bone of the underlying vertebral body (89), which prompted de Gauzy et al. (89) to propose avulsion-type “physeal” injuries as 2/3 of the morphological patterns that their Chance fracture classification system associates with flexion-distraction injuries of the pediatric spine. Fibers of both the NP and especially the AF insert themselves into the cartilaginous EP through an extensive branching of their embedded lengths, thereby achieving a highly effective anchorage within the cartilaginous EP (6, 8). Conversely, the articulation between the cartilaginous EP and vertebral body is considerably weaker than the integration achieved at the AF-EP interface. Experimentally, Berg-Johansen et al. (9) demonstrated that uniaxial tensile testing of human cadaveric inner IVD-EP-vertebrae extracts, resulted in failure at the EP-vertebra interface in 71% of their mechanical tests. Similarly, surgical (92) and postmortem (10) investigations of the human spine report that IVD failure at the EP-IVD interface was more commonly observed, even than tearing of the AF (87), and that increased grades of IVD degeneration carry a significantly greater probability of EP irregularity than non-degenerated IVDs, with the interface between the EP and vertebral bone being especially susceptible to disruption (10). Consistent observation (experimentally, surgically, and postmortem) of disruption and avulsion at the point of the articulation between the vertebra's cortical bone and the cartilaginous EP recommends that the vertebra-EP interface represents the mechanical weak link of the IVD-EP-vertebral body complex, when significant tensile/distraction forces are applied (9, 10, 92).

One additional mechanism pertinent to flexion-related injuries of the pediatric spine is child abuse. Skeletal fracture resulting from nonaccidental trauma in the pediatric has an estimated incidence of 1.54%, reported in the retrospective review of cases conducted by Zhao et al. (93) However, the true incidence of nonaccidental trauma in the pediatric population is difficult to ascertain, due to the significant social and legal ramifications surrounding such incidents. And so, while falls and MVCs are responsible for most pediatric fractures, Hobbs et al. (94) made an important observation when stating; “Most accidental fractures in infants and toddlers result from falls, although fractures are uncommon in falls of under a metre;” an important factor when assessing the proposed mechanism of injury responsible for pediatric fracture.

Numerous reports have demonstrated that young children/infants (i.e., <24–36 months of age), are significantly more likely to present with a skeletal fracture due to child abuse (including fractures of the spine), than older children (i.e., 10 + years of age) (93–103), recommending that future investigations related to the detection of nonaccidental musculoskeletal trauma in children should be tailored to specifically target this younger age group for further investigation.

No injury is pathognomonic in the identification of nonaccidental trauma in children, though importantly, multiple retrospective investigations (either case studies or larger cohorts of patients) attest to the moderate level of correlation between pediatric spine fractures, and nonaccidental injury (96, 104, 105). This pathognomy is bolstered when a child is still too young to walk, when there are multiple other signs of abuse (ranging from rib fractures to metaphyseal avulsions to ecchymosis to old and healing fractures, seemingly of different ages), and when there is a lack of sufficient history attributable to the magnitude of energy, required to generate a fracture (95, 99, 106–108). It must always be remembered when investigating nonaccidental trauma, that all fractures of the spine, unless there is an underlying pathophysiological condition responsible for weakness of the vertebrae [i.e., osteogenesis imperfecta, leukemia, immunosuppressant treatment (27), rickets, Menke's disease (109, 110)], represent a significant magnitude of energy involved in the abusive event.

Biomechanically, the significantly larger head to body proportion of infants, along with their significantly reduced muscle mass to body mass ratio, represent significant and contributive factors to the etiology of “shaken baby syndrome.” Etiologically, the biomechanics of “shaken baby syndrome” are typically represented as a rapid, forward-backward acceleration of an infant's torso, predominantly in the sagittal plane (101, 106, 108). Owing to the unique anatomic proportions of infants, the rapid shaking associated with such an event can generate significant and complex combinations of flexion, compression, extension, and distraction forces throughout the infant spine (101, 106–108). Anatomically, another uniqueness of the infant spine are the relatively horizontally oriented facet joints, especially throughout the cervical region (101, 106, 108). This horizontal orientation recommends that an infant's facet joints have a significantly reduced capacity to withstand horizontally orientated shear forces when such forces are imposed across the intervertebral space (101, 106, 108). “Shaken baby syndrome” therefore represents a complex kinematic event where the diminished capacity for the horizontally oriented facet joints to resist shear forces, paired with the large body proportions and small muscle mass of infants, suggests that other aspects of the spine's osteoligamentous anatomy are largely responsible for withstanding the forces imparted by the shaking event.

Fractures of the cervical spine due to abusive shaking of an infant are thought to manifest due to the interaction of the rapid acceleration forces imparted by the shaking event, with the significant inertia of an infant's large head, an infant's underdeveloped cervical musculature, and the infant's horizontally oriented facet joints; numerous case series have reported spine fractures and fracture-dislocations in infant patients, resulting from whiplash-like shaking events (98, 101, 106–108, 111–113).

The location and morphology of fracture most likely to manifest during abusive shaking, is a product of the specific forces imparted during the time of the event, though several consistent attributes have been noted with regards to these fractures. Younger children are more likely to suffer cervical spine injuries from an abusive shaking event than older children (101, 112, 113); in their review of the literature, Kemp et al. (101) made a distinction in injury patterns between “young” infants and “older” infants where younger infants (with a median age of 5 months) were more-often observed to suffer from injuries to the cervical spine region, while “older” infants (with a median age of 13.5 months) had a greater propensity for fractures to manifest in their thoracic and lumbar regions. Therefore, as children age and their head-torso proportions gradually transition toward that of an adult, this difference in body proportions seems to reduce the cervical spine's likelihood of being involved in an abuse-related shaking event (101, 112).

However, akin to the rapid forward-backward translation generated between an infant's head and torso across their cervical region, shaken baby syndrome also seems capable of manifesting as a thoracolumbar fracture of the spine. Kinematically, during shaking the mismatch in the inertias between accelerations of the infant's torso and pelvis, can result in a large propagation of mechanical forces in the axial direction, generating complex combinations of flexion, extension, tension, compression, and shear throughout the lumbar spine, all at a rapid velocity. In fact, a retrospective analysis performed by Jauregui et al. (98) reported the thoracolumbar region to have the highest abuse-related incidence of injury.

Perhaps the discrepancy in injury patterns reported in “young” infants vs. “older” infants is a product of an infant's developmental stage in relation to walking. Like the rest of the weightbearing components of the human skeletal system, an infant first learning to walk represents a significant mechanical stimulus, which encourages skeletal adaptation and remodeling. Prior to ambulation, the sagittal curvatures, the spine's state of calcification, and muscular development of the infant, is considerably different than post-ambulation. Therefore, Kemp et al.'s (101) differentiation between “younger” and “older” infants may represent an important biomechanical distinction in the musculoskeletal status of an ambulatory vs. non-ambulatory infant's spine. Numerous studies cite ambulation as a critical factor contributing to the injuries most likely to occur in the bones of the lower limbs (96, 102, 105), and though it remains to be demonstrated, it is not unreasonable to assume that the adaptive changes that occur in the pediatric skeleton in response to walking, may also underlie some of the differences in injury patterns of the pediatric spine, observed pre and post ambulation.

Compression fractures, growth plate (apophyseal) fractures/avulsions, and facet fracture/dislocations in the cervical, lumbar, and thoracolumbar regions of the spine, have all been associated through retrospective analysis, with “shaken baby syndrome,” (95, 98, 101, 103, 106–108, 111–113) where the rapid forward-backward acceleration of the torso and pelvis was proposed to enact complex patterns of flexion, extension, compression, shear, and longitudinal tension, across the cervical, lumbar, and thoracolumbar regions of the spine. However, somewhat counterintuitive to the proposed biomechanics above, the retrospective investigation conducted by Kleinman et al. (114), reported that 22/25 of the pediatric vertebral fractures they observed were compression fractures of the vertebral body, with one “Hangman's” fracture located at C2, and two sacral fractures in one child. Notably, Kleinman et al. (114) reported zero posterior element fractures and/or posterior element dislocations. As stated by Levin et al. (111), “The injury (thoracolumbar fracture with listhesis) is not produced by flexion alone. Rather, it is due to a combination of an axial load, flexion, and rotation.” Figure 2, Image 2ii, illustrates how rapid anterior-posterior translation can create regions of compression (on the concave side of the bending spine) and tension (on the convex side of the bending spine).

Despite significant heterogeneity in abuse-related injury fracture patterns of the pediatric spine, some commonalities do exist in the injury patterns recorded, and the physical mechanism most-likely for their manifestation. “Shaken baby syndrome,” especially when it results in injury to an infant's spine, is best described as a series of rapid back-and-forth translations, with the potential to impose complex kinematic patterns of loading. The forces imparted on the vertebrae, facet joints, and intervertebral discs of the spine may involve rapid intervals of flexion, extension, compression, shear, and tension, generating complex combinations of forces throughout the shaken patient's spine [i.e., the “S” shaped kinematics identified by Panjabi et al. (86) during simulated rear-end MVCs investigating cervical “whiplash”]. Depending on the precise character of the mechanical forces imparted, the vertebral body, cartilaginous EP, facet joints, longitudinal ligaments, and even the spinal cord in isolation, may be injured during the abusive event.

“Shaken baby syndrome” is a difficult biomechanical mechanism to investigate. As it pertains to pediatric injuries of the vertebral body, the anatomical site most-likely to fracture during “shaken baby syndrome” likely relates to an infant's relative body proportions, the maturity of the spinal column at the time of the event, the capacity for the patient to ambulate, and the specific combination of forces imparted on the spine by the incident. While all cases of child abuse are terribly unfortunate, clinicians and researchers must be familiar with the clinical and morphological presentation of such injuries, in order that the cause of such an injury not be overlooked; failure to appropriately identify abuse-related fractures, has potentially catastrophic and lifelong consequences for the patient involved (27, 94, 98, 107, 114–116).

## 4 Vertebral fractures in the adolescent spine

### 4.1 Contributing factors to the vertebral fracture process

The vertebrae of the human skeleton grow in both diameter and length through a coordinated expansion of over 130 active growth plates (1). The vertebral body lacks the true epiphyses of normal long bones due to the cartilaginous EPs articulating with the IVD at a vertebra's cranial and caudal aspects. However, longitudinal growth is still a product of coordinated endochondral ossification of the vertebral bone beneath these cartilaginous EPs (3, 4, 117, 118).

The vertebral apophyses represent an interesting structure, as each vertebral body possesses two apophyseal rings (one superior and one inferior), each of which wrap circumferentially around the vertebral body. While endochondral ossification of the vertebral body is responsible for longitudinal growth, appositional growth of the ring apophyses increases vertebral body diameter (117–119). Skeletal maturity is defined as fusion of the growth apophyses with the vertebral body, such that these regions fully ossify, and become indistinguishable from the bone in their vicinity (117–119). Prior to ossification, increases in vertebral body length and diameter are still possible, but once ossification has occurred, vertebrae will have reached their adult dimensions (117–119). Longitudinal growth of vertebral bodies is generally complete between 11 and 16 years of age in females and 12–16 years in males (1). Similarly, Costa et al. (120) reported that ossification of the spine's ring apophyses occurs between 7 and 15 years of age in females and 9–15 years in males, with fusion following (13–19 years of age for females; 14–19 years in males). However, chronological age and skeletal maturity are not synonymous, and unfused ring apophyses have been documented radiographically in patients up to 25 years of age (121).

The biomechanics of vertebral fracture in the adolescent spine seem to correlate with the interaction of biomechanical forces with the “weak link” of the unfused apophyses. Due to being surrounded by a considerably stiffer bony matrix, the relatively softer cartilage of the unfused apophyses create significant stress concentrations, marking these as the sites of greatest vulnerability to fracture, in the skeletally immature vertebral body.

In a study by Karlsson et al. (122), compression of 16 human cadaveric motion segments (mean age of 16.3 years) at a rate of 5 mm/min resulted in rupture of the cartilaginous EP in 9 of the 16 specimens and separation of the EP from the vertebra in 12 of the 16 specimens, highlighting the vulnerability of the cartilaginous EP in this age group. Similarly, a series of case reports on pediatric flexion-distraction injuries found that these injuries manifest as “physeal” injuries (damage to the unfused apophyseal growth plates) rather than IVD injuries; these observations concur with hypotheses that the cartilaginous growth plates are structurally weaker than the healthy IVD (123).

Another important feature of the adolescent spine is the state of hydration of its NP. During axial compression, the NP's ability to increase its hydrostatic pressure is critically linked to supporting the AF against buckling and internal collapse, as demonstrated by nuclectomy experiments of motion segments (124). As such, the process of vertebral fracture as it relates to the human spine is also highly correlated with the load-sharing mechanisms within the IVD. The reduced stiffness of the unfused apophyseal cartilage appears to represent a critical component regarding the mechanism of fracture that will be generated in the adolescent spine.

Adolescents can incur similar vertebral fracture injuries to infants/children during falls from height and MVCs, but unlike infants and young children, activities of daily living, engagement in sporting activities (especially elite-level sport), and occupational exposures also contribute to the generation of vertebral fractures in the adolescent age group. Burst fractures, and Chance and cartilaginous EP avulsion-fractures can be generated in the adolescent spine during falls from height or MVCs, with similar biomechanical processes to the infant spine, except that adolescents possess a head-torso ratio and muscle mass-body mass ratio more comparable to adults. Fractures generated by acute, and repetitive mechanical insults will be outlined in the following sections.

### 4.2 Biomechanics of vertebral fracture in the adolescent spine

#### 4.2.1 Unfused apophyseal seams, accumulated hydrostatic pressure in the nucleus pulposus, and endplate concavity

In the adolescent spine, the biomechanics of vertebral fracture appear to be critically linked to the pressurization response of a hydrated NP, in conjunction with the unfused cartilaginous seams of the apophyses. During axial loading when the vertebral bodies translate towards one another, the central NP is compressed. In adolescence the NP of the IVD is abundantly populated with proteoglycans, making this region quite hydrated (39–42). Compression increases the hydrostatic pressure of the NP resulting in a viscoelastic stiffening effect.

In a series of *in vitro* experiments using porcine cervical models to represent the human adolescent spine, EP fractures were produced with rapid-rate pressurization of the NP (125–128). Similarly, a finite element model of the human spine constructed by Fields et al. (74) reported that the Poisson's ratio achieved by the NP of the loaded IVD directly related to the location and magnitude of tensile strain generated in the proximal EPs. In addition, Farrell and Riches (54) report the Poisson's ratio of the NP to be strain-rate dependent, further illustrating its viscoelastic mechanical response.

Therefore, under scenarios of rapid axial compression, viscoelastic stiffening will result in a relatively incompressible NP. Because of this, the compressed vertebral bodies bend around the incompressible NP causing increased flexural stress across the EPs of the vertebrae. In the adolescent spine, the unfused cartilaginous seams of the vertebral apophyses represent a significant stress concentrator, thereby creating a weak point. Experiments employing axial compression/impact to adolescent animal models of the spine (129–137) report the unfused cartilaginous apophyses as the site most vulnerable to fracture; a trend that was consistent across multiple loading paradigms including slow velocity compression (135, 137), dynamic compression (134), as well as rapid intradiscal pressurization (125–128). Additionally, even when motion segments were held under flexion or extension and then compressed, the vertebra's unfused apophyses remained the most susceptible region to injury in motion segments (131).

Small, sub-critical fractures can also be generated in the adolescent spine due to the pressurization-related response of the NP. Schmorl's nodes can be conceptualized as miniature EP fractures where focalized perforations of the cartilaginous EP occur due to an excessive accumulation of hydrostatic pressure. Importantly, Schmorl's nodes only impact a very small cross-section of the vertebral body, appearing on MRI as “vertical herniations” where a small “node” of the NP intrudes into a focalized pocket of the cartilaginous EP. Until recently, Schmorl's nodes have been considered of “benign” character, and only an incidental finding during radiography and MRI. However, a growing body of human and animal-model research attests to the relationship between interruptions to the structure/function of the cartilaginous EP and degeneration of the IVD (138–141); this relationship has been further suggested to be dose-dependent (141). As a result, the biomechanics of the hydrated, adolescent NP may represent a critical factor toward the initial development of conditions favourable to degeneration of the IVD. One additional note is that, while degeneration of the IVD has a strong, positive association with chronological age, increasing concern about the relationship between IVD and EP health during adolescence may be warranted because, although adolescent humans present with a low frequency of IVD degeneration, the weak point represented by the cartilaginous seams of the unfused vertebral apophyses, may represent a significant vulnerability toward EP injury during this period of the human lifespan.

While the apophyseal seam represents a mechanical vulnerability for all spines prior to skeletal maturity, the curvature of the vertebral EPs varies substantially during the years of skeletal growth, which also has a significant impact on EP fracture mechanics. In an *in vitro* experiment, rabbit motion segments from the T10/T11-L5/6 levels were exposed to rapid loading (1.95 m/s) resulting in EP fractures in 15 of the 16 specimens. The authors noted higher failure loads in specimens with larger and deeper EP curvatures (21). Furthermore, retrospective investigation of human patients treated for NP herniation reported that EPs rated as “flat” or “irregular” were between 3.3 and 4.3 times more likely to correlate with symptoms of pain, than EPs rated as “concave.” (142) Last, radiographic investigation of human tissue removed during surgery (142) reported that vertebral EP morphology appears to be directly related to the degeneration status of the adjacent IVD (142, 143). As such, both human and animal research suggest that deeper EP concavity may represent a protective effect against EP fracture/deformity.

Overall, maturity of the vertebral apophyses, the viscoelastic properties of the adjacent NP, and the concave morphology of an EP, appear to represent variables that significantly contribute to the biomechanics of vertebral body fracture in the adolescent spine.

### 4.3 Repetitive sport-related extension

#### 4.3.1 Ring apophysis fracture (RAF)

The vertebral apophyses begin to calcify at a mean age of 11 years (119, 120), while complete ossification is not complete until a mean of 18 years, with reports of unfused vertebral apophyses persisting to the age of 25 (121). Therefore, the unfused vertebral apophyses represent a significant point of vulnerability in the adolescent vertebral body, where either acute overload or repetitive microtrauma can result in vertebral fracture at this site. Furthermore, during compression plus spine extension, biomechanical contributions from the spine's facet joints appear to play a significant role in the axial compression mechanics of the spine (144), which may have important contributions toward the etiology of ring apophysis fractures (RAFs); this relationship will be discussed in Section 4.4.

RAFs are not a common clinical entity, representing only 5.8% of all surgeries associated with lumbar IVD herniation (145). Small case series reports, and retrospective analysis of the treatment of patients afflicted with RAFs, represent the bulk of the current knowledge regarding this clinical entity. Adolescents are perhaps the most well-documented cohort with regards to RAF etiology, with multiple case series examining the potential for RAFs to develop in the skeletally immature spine (145–149), while additional concern surrounds the development of RAFs in elite-level athletes during adolescence due to the high demands of training and competition (149–153).

**Figure 3:**
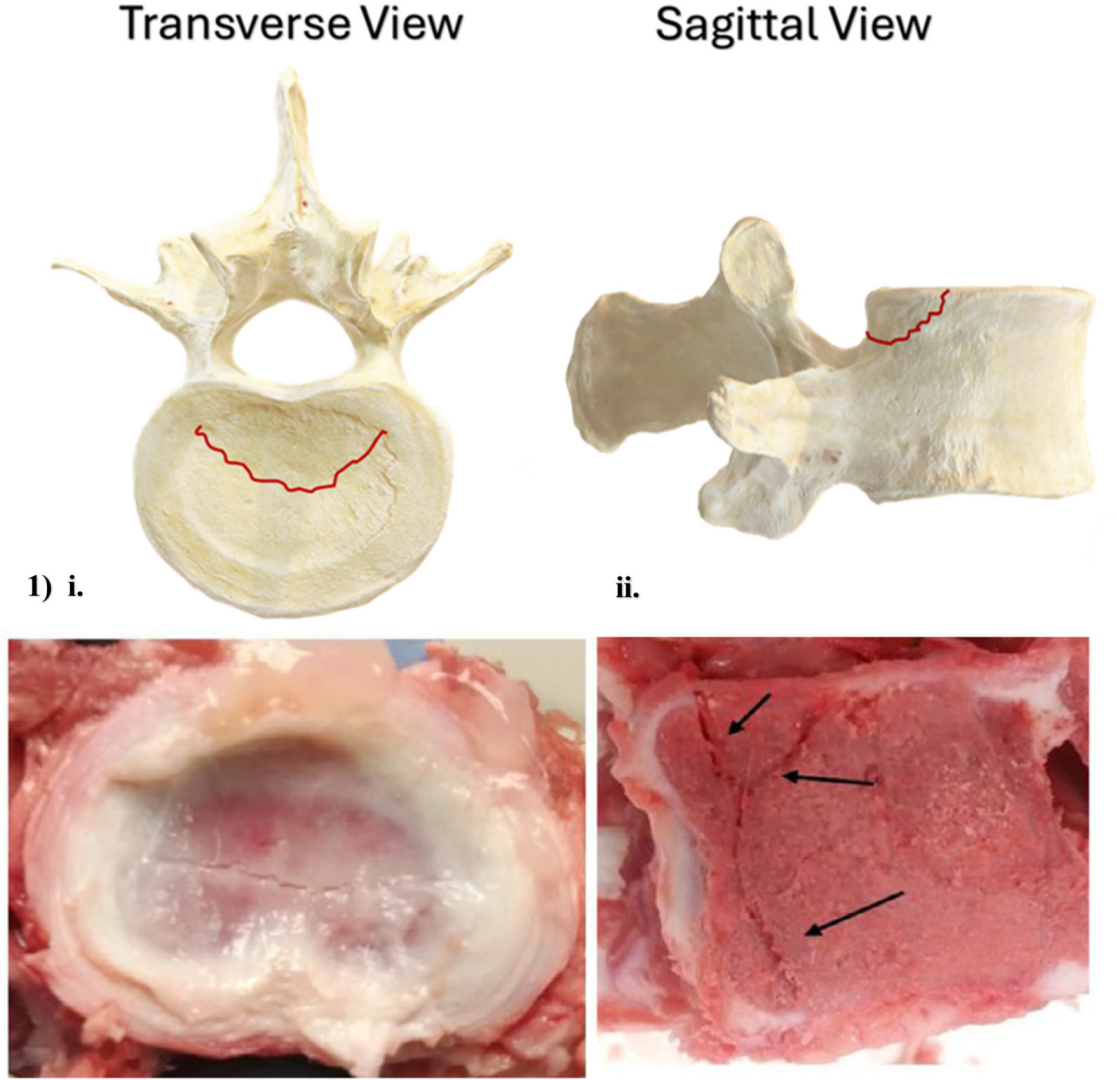
Axial and sagittal schematic, and axial and sagittal images of a porcine cervical vertebra, illustrating most common ring apophysis fracture morphology. Porcine cervical fracture photographs are reprinted with permission (132).

Numerous retrospective analyses of RAF diagnosis, and both conservative and surgical treatments, concur that when high physical demands are imposed upon a skeletally immature spine, the cartilaginous seams of the apophyses represent its site of greatest vulnerability (145, 147, 154–161). MRI, CT and plain radiography have all visualized RAFs, with the most common morphology appearing as a crescent-shaped fracture avulsing from the posterior or posterolateral aspect of the vertebra's inferior or superior apophysis when observed in the transverse plane (Figure 3, Image 1, i), and as a “corner fracture” in the sagittal plane (Figure 3, Image 1, ii). In addition, researchers note a high frequency of NP herniation in RAF patients, suggesting that separation of the apophyses may represent an EP-mediated mode of IVD herniation, capable of initiating Modic changes (162), or potentially relating to subsequent IVD degeneration (92).

Little in the way of *in vitro* experimentation has been done to document RAF etiology, but an experiment performed by Corbiere et al. (163) demonstrated that repetitive flexion of a cervine spine model resulted in avulsion of the growth plate, morphologically similar in appearance to RAFs documented juvenile human spines. Similarly, an *in vitro* investigation employing a porcine cervical model of the spine performed by McMorran and Gregory (137) demonstrated that acute overload of motion segments via axial compression consistently resulted in EP fractures bearing a strong RAF-like morphology. *In vitro* research using skeletally immature animal models in lieu of human tissue consistently report the apophyses as the site of greatest mechanical vulnerability (131–137), recommending that this mechanical vulnerability persists across multiple different mammalian spines throughout the period of vertebral body growth.

Pediatric IVD herniation is considerably less common than herniations affecting adult patients, with a reported incidence of hospitalization for children and adolescents ranging from 0.1% to 0.2%, (148, 164) and an incidence of surgery related to IVD herniation of 1.2% (25/1,750 patients) (151). Conversely, IVD herniation affects 2%–3% of adults over the age of 35 (165). Despite its infrequency, pediatric IVD herniation possesses a considerably greater association with a precipitating mechanical trauma than herniation of the adult IVD. Estimates vary, but studies consistently report that 30%–60% of pediatric IVD herniation patients can point to a specific mechanical insult as being responsible for their injury (148).

The strong association between IVD herniation and RAF in adolescents suggests that the NP is more likely to herniate through the unfused apophysis rather than the AF's layers. In their case series of 24 patients with lumbar IVD herniation who underwent µ-CT imaging, Chen et al. (149) found that 42% (10/24) also had an accompanying RAF. Similarly, in their retrospective review of 31 patients <21 years old who received laminotomy surgery to treat IVD herniation, Banerian et al. (152) reported that 19% (6/31 cases) also had an accompanying avulsion fracture of the vertebral EP (i.e., RAF). Further, these authors reported 2 additional cases where IVD herniation was accomplished via burst fracture of the vertebral body.

However, this potential apophyseal-mediated mode of IVD herniation has not been consistently demonstrated in the literature. Ozgen et al. (150) reported that 82% (14/17) of adolescent IVD herniation patients attributed their injury to a specific traumatic event; however, among those who underwent µ-CT imaging (*n* = 8), none exhibited an accompanying vertebral fracture.

The strong association between trauma and IVD herniation in the pediatric population, paired with consistent observations of the mechanical vulnerability of the spine's unfused vertebral apophyses, highlights the need for further research. Future studies should explore whether these unfused regions serve as structural weak points that contribute to RAFs, apophysis-mediated IVD herniation, Modic changes, and premature degeneration of the IVD.

### 4.4 Contribution of the facet joints to vertebral fracture mechanics, and the Tokushima theory of growth plate slippage

Pure anterior shear forces (i.e., those responsible for anterolisthesis of one vertebral body over another) are largely resisted by the facet joints. In an experiment performed by Raynor et al. (166), resection of more than 70% of cervical human cadaveric spine segments' facet joints resulted in a mean failure load of 159.0 lbs, whereas facet joints with less than 50% of their height resected, failed at a significantly higher mean load of 208.0 lbs. The degree of shear-resistance is further influenced by the orientation of the facet joints such that more coronal orientation will increase the magnitude of anterior-posterior shear resistance imparted by these joints (167, 168).

Extension also influences facet joint loading. Biomechanically, during extension the space between facet joints is reduced, thereby increasing compression (144, 169–171). A finite element model of a lumbar motion segment constructed by Yang and King (144) reported that with 10 mm posterior application of axial load for non-degenerated IVDs (creating a slight posterior extension moment), the facet joints carry a mean of 17.5% of the axial load applied to a motion segment, and that with degeneration of the IVDs (i.e., significant reduction of IVD height), the axial load endured by the facet joints can rise to as high as 25.8% (144). Compression acting through the facet joints results in significant load transmission through the pars interarticularis, across the pedicles, into the posterior vertebral body (172, 173). During excessive compression plus extension of the spine, the pars interarticularis represents the initial point of failure in the posterior column of the spine. Initial microfractures tend to manifest in this region, in the inferior-to-superior direction, due to the concentration of bending and torsion forces at the inferomedial aspect of the pars interarticularis (172, 173). Upon failure of the pars interarticularis, compressive forces propagate into the posterior of the vertebral body, which now lacks mechanical support from the damaged posterior column. As demonstrated in the Tokushima theory of growth plate slippage (representing an etiology of injury regarding chronic spine loading), once a lesion to the pars interarticularis occurs, the motion segment's kinetics are pathologically altered, concentrating additional mechanical forces onto the vertebral apophyses (173, 174). Due to the inherent weakness of the apophyses' growth cartilage compared to the cancellous and cortical bone of the vertebral body, the concentration of compression forces onto the unsupported posterior vertebral body interacts with the unfused, cartilaginous seams of the vertebra's ring apophysis; the “weak point” of the skeletally immature spine (131–137, 145, 147, 154–161). Failure of the pars interarticularis from excessive compression plus extension movements of the spine subsequently makes the unfused vertebral apophysis more vulnerable to fracture via compressive overload, which most commonly manifests as a RAF.

Ikata et al. (175) reported that following a pars interarticularis lesion and subsequent RAF, anterior translation and listhesis of the injured vertebral level was most susceptible during the stage of adolescent growth. A more recent investigation demonstrated that patients in their active growth stage have the highest incidence of anterior vertebral listhesis [80% Sairyo et al. (176)], while those defined as being in the “late” growth stage saw anterior listhesis in only 11% of lesions (176). Most importantly, once the cartilaginous apophyses had fused, no patients were observed to suffer anterior vertebral listhesis (176).

The differentiating mechanism as to whether RAF or growth-plate-mediated anterior listhesis manifests in the adolescent spine has not been fully elucidated, but the degree of ossification of the apophyseal cartilage seems to interact with the type of compression and extension forces experienced by the spine, under both acute and chronic scenarios of loading. Therefore, the skeletal maturity of the spine that experiences compression in combination with extension and/or lateral bending and/or twisting movements, is likely a significant contributing factor to the etiology of any resulting vertebral fracture (176).

## 5 Conclusion

Complex, three-dimensional loads can be placed on the human spine at any age, but the significantly different body proportions and muscular competency of infants, a hydrated IVD, and the presence of numerous unfused growth plates prior to skeletal maturity, represent significant variables that contribute to the biomechanics of pediatric spine fractures.

Vertebral fractures are a severe injury at any age with the potential to develop multiple comorbidities and significantly reduce a patient's quality of life. However, the mechanical processes underlying the injury patterns observed in the infant and adolescent spine present with several unique mechanical concerns not found in the spine after skeletal maturity.

### 5.1 Future directions

Due to the infrequency of pediatric vertebral body fractures in regular clinical practice, and the scarcity of post-mortem investigations available for this age group, literature on these injuries is less extensive than the literature pertaining to fractures in the adult and elderly spine. Several key domains of pediatric spine injury remain relatively underexplored, such that considerable advances to the fields of clinical and surgical care may result from a better understanding of different facets of the injury and disease processes, relevant to this age group. As such, the following represent several potentially important questions for the field of pediatric vertebral fracture research, where an improved understanding of the underlying biomechanical relationships has the potential to benefit both clinical and surgical treatment options for future vertebral fracture patients.

1) How does vertebral EP morphology (i.e., more or less concaved) interact with a vertebral body's vulnerability to fracture?
2) How does the natural loss of the NP's proteoglycans interact with the hydrostatic and viscoelastic mechanisms relating to vertebral and EP fracture?
3) How much weaker is the unfused apophysis than the surrounding vertebral bone? Can future experiments begin to quantify the magnitude of this mechanical disparity?
4) How do pediatric diseases (i.e., leukemia) interact with the biomechanical relationships surrounding vertebral fracture? What additional prevention and treatment considerations would optimally benefit these patients?
